# Prevalence of central serous chorioretinopathy in Denmark

**DOI:** 10.1111/aos.17520

**Published:** 2025-05-11

**Authors:** Ida N. Frederiksen, Danson V. Muttuvelu, Rodrigo Anguita, Lasse J. Cehofski, Nathalie S. Eriksen, Carsten Faber, Mads K. Falk, Lorenzo Ferro Desideri, Giuseppe Giannaccare, Jakob Grauslund, Michael Stormly Hansen, Josef Huemer, Morten B. Larsen, Ligor P. Kiruparajan, Chris B. Søndergaard, Andrea Taloni, Tobias E. Torp‐Pedersen, Elon H. C. van Dijk, Marie L. R. Rasmussen, Yousif Subhi

**Affiliations:** ^1^ Department of Ophthalmology Rigshospitalet Glostrup Denmark; ^2^ MitØje v/ Danske Speciallæger ApS Aarhus Denmark; ^3^ Faculty of Health and Medical Sciences University of Copenhagen Copenhagen Denmark; ^4^ Moorfields Eye Hospital NHS Foundation Trust London UK; ^5^ Department of Ophthalmology, Inselspital University Hospital Bern, University of Bern Bern Switzerland; ^6^ Department of Ophthalmology Aalborg University Hospital Aalborg Denmark; ^7^ Department of Ophthalmology Zealand University Hospital Roskilde Denmark; ^8^ Department for Bio Medical Research University of Bern Bern Switzerland; ^9^ Bern Photographic Reading Center, Inselspital University Hospital Bern Bern Switzerland; ^10^ Eye Clinic, Department of Surgical Sciences University of Cagliari Cagliari Italy; ^11^ Department of Ophthalmology Odense University Hospital Odense Denmark; ^12^ Department of Ophthalmology Vestfold Hospital Trust Tønsberg Norway; ^13^ Department of Clinical Research University of Southern Denmark Odense Denmark; ^14^ Department of Ophthalmology and Optometry Kepler University Hospital, Johannes Kepler University Linz Austria; ^15^ Øjenlæge Morten Bøgelund Larsen Tønder Denmark; ^16^ Department of Translational Medicine University of Ferrara Ferrara Italy; ^17^ Department of Ophthalmology Ospedali Privati Forlì “Villa Igea” Forlì Italy; ^18^ Istituto Internazionale per la Ricerca e Formazione in Oftalmologia Forlì Italy; ^19^ Department of Ophthalmology Leiden University Medical Center Leiden The Netherlands; ^20^ Rotterdam Eye Hospital Rotterdam The Netherlands

**Keywords:** central serous chorioretinopathy, Denmark, epidemiology, prevalence

## Abstract

**Purpose:**

Central serous chorioretinopathy (CSC) is a prevalent maculopathy, but epidemiological studies are few. In this study, we determined the prevalence of CSC for the first time in a Scandinavian population.

**Methods:**

This cross‐sectional study was based on nationwide opportunistic retinal examination from 79 high street chain optician stores in Denmark. Retinal imaging was made using non‐mydriatic colour fundus photography. Any abnormal result in the optometrist‐facilitated retinal examination was referred to tele‐ophthalmologic evaluation, which was performed by experienced consultant ophthalmologists who diagnosed CSC.

**Results:**

During the 4‐year study period, a total of 968 610 unique individuals underwent retinal examination, which corresponded to 16.3% of the entire population of Denmark. Of these, 113 individuals were diagnosed with CSC, which corresponded to a prevalence of 14 per 100 000. Individuals with CSC presented at a mean age of 48.2 ± 12.3 years; however, CSC was present in a large age range as both teenagers and the elderly with CSC were identified. Male biological sex was a statistically significant risk factor (odds ratio: 2.33; 95% confidence interval: 1.64–3.33, *p* < 0.0001). By extrapolating prevalence numbers to population statistics, we estimate that 219 females and 511 males had CSC in Denmark in December 2022.

**Conclusion:**

We identified a prevalence of 14 per 100 000, confirmed male biological sex as a significant risk factor for CSC, and found that the disease most commonly occurs among individuals aged 30–60 years. Further studies with multimodal imaging including optical coherence tomography are warranted for better accuracy.

## 1 INTRODUCTION

Central serous chorioretinopathy (CSC) is considered the fourth most prevalent exudative maculopathy after age‐related macular degeneration (AMD), diabetic maculopathy and retinal vein occlusion (Feenstra et al., 2024). The pathophysiology of CSC is considered to be the consequence of choroidal congestion and arteriovenous anastomoses, which drive serous fluid out of the choroidal vessels, through Bruch's membrane and the retinal pigment epithelium, and into the subretinal space (Brinks et al., 2022; Cheung et al., 2019; Feenstra et al., 2024). The disease is popularly classified as either acute CSC or chronic CSC (Feenstra et al., 2024). Acute CSC is defined as a recent onset of the disease, and the majority of these cases will experience spontaneous resolution of the subretinal fluid (Daruich et al., 2017). Chronic CSC is typically defined as the presence of the disease for at least 6 months without spontaneous resolution of subretinal fluid, and these cases typically need photodynamic therapy to prevent photoreceptor atrophy, decreased vision‐related quality of life and irreversible vision loss (Breukink et al., 2016; Peiretti et al., 2015; van Dijk et al., 2023; Wang et al., 2002).

Despite the relatively high prevalence of CSC, epidemiological studies based on retinal imaging are few and individual studies are small in size (Liew et al., 2013). To our best knowledge, no evidence currently exists on the prevalence of CSC in a Nordic/Scandinavian population. In this study, we evaluated the prevalence of CSC in Denmark by using data from a nationwide optometrist‐based retinal examination system (Muttuvelu et al., 2021; Rasmussen et al., 2024). Throughout 79 optician chain stores (Louis Nielsen, Specsavers), customers received non‐mydriatic colour fundus 45° photography in relation to the sale of optical products, adjustment of glasses or subjective experience of visual disturbance. Any potentially abnormal results were uploaded to a tele‐ophthalmologic service, where experienced consultant ophthalmologists provided a diagnosis and plan for the customer. Here, we report on the prevalence of CSC using this opportunistic nationwide examination strategy for all customers examined in the period from 2019 to 2022.

## 2 METHODS

Optometrist‐facilitated retinal examination was performed across all of Denmark through 79 high street optician stores of Louis Nielsen (Louis Nielsen A/S, Aalborg, Denmark). All involved optometrists underwent systematic training. The optometrist collected a brief history, and measured refraction, best‐corrected visual acuity, intraocular pressure, and performed a basic slit‐lamp examination, 4‐in‐1 visual field report, and retinal imaging with colour fundus 45° photography using CenterVue DRS (iCare, Helsinki, Finland) or DRS plus (iCare, Helsinki, Finland), and in some cases 4‐in‐1 visual field report. The optometrist reviewed the results, and any abnormalities or suspected abnormalities would lead to a referral to a tele‐ophthalmological system for evaluation by at least one experienced ophthalmologist. The diagnosis of CSC was at the ophthalmologists' discretion based on available information and fundus photography. The group of ophthalmologists was able to discuss cases between each other. In case of an abnormality, the ophthalmologist at the teleophthalmology system would refer to further examination through the publicly funded healthcare system in Denmark. The details of the organization of this service are published in more detail in previous studies (Muttuvelu et al., 2021; Rasmussen et al., 2024).

This was a cross‐sectional study of referred abnormal cases from the high street optician stores to the tele‐ophthalmological service within the time period from 1 January 2019 to 31 December 2022. We extracted data on those examined and those who obtained a diagnosis of CSC. Thus, our dataset simply consisted of the diagnosis of CSC (Yes/No) and demographic data (age and biological sex) for all unique individuals examined through this study. Only the first examination was included, that is, if the customer was examined again, repeated data were not included for this study. This study followed the tenets of the Declaration of Helsinki. Prior to any data sharing, all patients provided mandatory consent to data processing and company policies. All data storage and data management aspects complied with the standards of European Union General Data Protection Regulation. All data extracted were fully anonymous and classified as quality assessment; hence, neither ethics approval nor any permission for the use of the data were needed according to The Danish Data Protection Agency.

Summary data were reported on age and biological sex of those examined throughout the 4‐year study period and of those diagnosed with CSC. We calculated confidence intervals for the prevalence estimates using the Wilson score interval with finite population correction adjustment. All statistics were made using Microsoft Excel for Mac (version 16.81). Statistical significance was defined as *P* values below 0.05.

## 3 RESULTS

Throughout the 4‐year study period, a total of 968 610 unique individuals underwent retinal examination. This corresponded to 16.3% of the entire population in Denmark (5 934 002 in December 2022 (Statistics Denmark, 2025)). Distribution of the examined individuals according to decades of age and biological sex, and the proportion within the demographic strata of the Danish population that they represent are presented in Table 1. The lowest proportion of the population examined was in the age categories of 0–10 years and 101+ years, in which, respectively, 0.3% and 2.9% of the population were examined. The greatest proportion of the population examined was in the age categories 41–50 years, 51–60 years, 61–70 years and 71–80 years, with, respectively, 22.9%, 24.6%, 24.3% and 23.5% of the population strata. A higher proportion of the female population compared to the male population underwent examination (18.2% vs. 14.4% for biological females vs. biological males). [Correction added on 21 September 2025, after first online publication: The percentage of the population examined has been corrected in the preceding paragraph.]

**TABLE 1:** Number of unique individuals examined, population of Denmark and the percentage of the entire population of Denmark examined within the 4‐year study period.

Age	Individuals examined			Population of Denmark			Percentage of population examined		
	Females	Males	Total	Females	Males	Total	Females	Males	Total
0–10	1357	855	2212	330 437	349 133	679 570	0.4%	0.2%	0.3%
11–20	45 944	27 279	73 223	336 293	351 609	687 902	13.7%	7.8%	10.6%
21–30	60 901	41 241	102 142	391 758	406 319	798 077	15.5%	10.1%	12.8%
31–40	51 782	33 487	85 269	356 083	367 642	723 725	14.5%	9.1%	11.8%
41–50	98 276	72 291	170 567	372 224	371 085	743 309	26.4%	19.5%	22.9%
51–60	106 949	91 715	198 664	402 097	404 109	806 206	26.6%	22.7%	24.6%
61–70	86 383	75 889	162 272	340 353	328 058	668 411	25.4%	23.1%	24.3%
71–80	69 345	63 413	132 758	298 792	266 561	565 353	23.2%	23.8%	23.5%
81–90	20 228	18 298	38 526	131 252	94 808	226 060	15.4%	19.3%	17.0%
91–100	1779	1177	2956	24 638	10 029	34 667	7.2%	11.7%	8.5%
101+	19	2	21	636	86	722	3.0%	2.3%	2.9%
Total	542 963	425 647	968 610	2 984 563	2 949 439	5 934 002	18.2%	14.4%	16.3%

**FIGURE 1:**
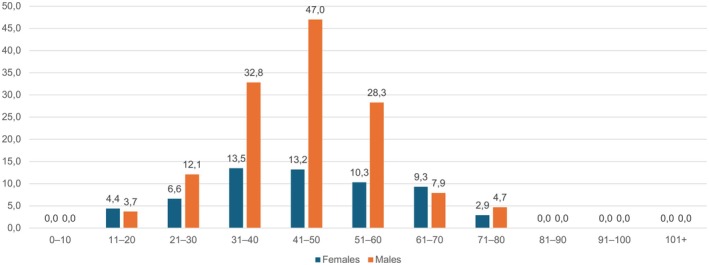
Distribution of the prevalence of central serous chorioretinopathy by age and biological sex. [Correction added on 21 September 2025, after first online publication: Figure 1 was revised.]

Throughout the study period, 133 unique individuals were diagnosed with CSC in one or both eyes (Table 2). These individuals constituted 14 per 100 000 individuals examined. Age was 48.2 ± 12.3 years, and 86 (64.7%) were of male biological sex. Male biological sex was a statistically significant risk factor for prevalent CSC (odds ratio: 2.33; 95% confidence interval: 1.64–3.33, *p* < 0.0001). Detailed distribution of the prevalence according to age strata and biological sex is presented in Figure 1.

**TABLE 2:** Prevalence of central serous chorioretinopathy (CSC). [Correction added on 21 September 2025, after first online publication: Table 2 was revised.]

Age	Number of cases with CSC			Prevalence in examined individuals, cases per 100 000 (95%CI)		
	Females	Males	Total	Females	Males	Total
0–10	0	0	0	0.0 (0.0–281.1)	0.0 (0.0–446.2)	0.0 (0.0–172.8)
11–20	2	1	3	4.4 (1.3–14.6)	3.7 (0.7–19.6)	4.1 (1.5–11.4)
21–30	4	5	9	6.6 (2.7–15.7)	12.1 (5.4–27.2)	8.8 (4.8–16.1)
31–40	7	11	18	13.5 (6.9–26.5)	32.8 (18.8–57.3)	21.1 (13.7–32.5)
41–50	13	34	47	13.2 (8.3–21.0)	47.0 (34.8–63.5)	27.6 (21.5–35.4)
51–60	11	26	37	10.3 (6.2–17.0)	28.3 (20.3–39.7)	18.6 (14.1–24.6)
61–70	8	6	14	9.3 (5.1–16.7)	7.9 (4.0–15.7)	8.6 (5.5–13.6)
71–80	2	3	5	2.9 (0.9–9.1)	4.7 (1.8–12.3)	3.8 (1.8–8.0)
81–90	0	0	0	0.0 (0.0–16.1)	0.0 (0.0–16.9)	0.0 (0.0–8.3)
91–100	0	0	0	0.0 (0.0–199.9)	0.0 (0.0–287.3)	0.0 (0.0–118.7)
101+	0	0	0	0.0 (0.0–16419.5)	0.0 (0.0–65495.0)	0.0 (0.0–15099.7)
Total	47	86	133	8.7 (6.7–11.2)	20.2 (16.6–24.6)	13.7 (11.8–16.0)

Abbreviations: 95%CI, 95% confidence interval; CSC, central serous chorioretinopathy.

Based on the prevalence estimates and the population numbers, it can be extrapolated that 219 females and 511 males had CSC in Denmark as of December 2022.

## 4 DISCUSSION

In this study, we present the results of the largest‐to‐date prevalence study of CSC in a European population and the first prevalence study of CSC in a Nordic/Scandinavian population. Based on optometrist‐assisted tele‐ophthalmological nonmydriatic fundus photography examination of 968 610 individuals in Denmark, which corresponds to 16.3% of the entire population, we are able to provide important epidemiological data for the fourth most common exudative maculopathy. These numbers also shed light into the gravity of the recent global verteporfin shortage, which left a large number of patients globally without the gold standard treatment (Feenstra et al., 2024; Sirks et al., 2022; Sirks et al., 2024).

We report the prevalence of CSC to be 14 per 100 000 individuals based on optometrist‐assisted tele‐ophthalmological nonmydriatic fundus photography examination. Intuitively, based on our clinical experience, this seems to be a low number. Indeed, studies using various health insurance claims or treatment databases indicate a significantly higher number of patients: Kido et al. (2022) from Japan reported the annual incidence of CSC to be 34 per 100 000 individuals, Rim et al. (2018) from South Korea reported a similar annual incidence rate of 35 per 100 000 and Tsai et al. (2013) from Taiwan reported an annual incidence rate of 21 per 100 000. Considering that these are incidence rates rather than prevalence rates, one would expect a much higher prevalence rate in a prevalence study. These differences highlight the challenges of large‐scale epidemiological investigations of CSC. On the one hand, fundus photography may not allow for accurate diagnosis of all cases of CSC, especially when subtle, when in remission or in complex cases with the development of atrophy or macular neovascularization. Thus, a fundus photography‐based approach without utilizing optical coherence tomography is at risk of underdiagnosing a number of cases. On the other hand, a registry‐ or insurance claims‐based approaches are at risk of overdiagnosing CSC, as a wide range of retinal conditions can present with subretinal fluid and are differential diagnoses of CSC (van Dijk & Boon, 2021). Sometimes, registering a patient with CSC may be the least incorrect option when there is a subretinal fluid or a patient receives photodynamic therapy for any reason (van Dijk et al., 2020).

Kitzmann et al. (2008) reported the incidence of CSC in Olmsted County in Minnesota, United States, in the period 1980–2002, in a predominantly White population. Here, the annual incidence of CSC was reported to be 5.8 per 100,000, which is significantly lower than that reported for Asian populations. This study was based on patient records in Olmsted County provided by the Mayo Clinic and the Olmsted Medical Group, and therefore based on patients with symptoms, who sought medical care and were seen in one of the affiliated hospitals. Although an annual incidence of 5.8 per 100,000 may be higher than the prevalence estimate in our study, the incidence rate in Olmsted County was significantly lower than that in Asian populations (Kido et al., 2022; Rim et al., 2018; Tsai et al., 2013). Reports have speculated that the incidence may be higher in Asians than in Whites, and that incidence rates in Whites may be higher than in Africans and Blacks (Fung et al., 2023). Studies which have investigated potential differences across ethnicities provide conflicting results, and evidence suggests that at least some of these differences may be due to CSC being underestimated in some population groups (Desai et al., 2003; Mehta et al., 2018).

The mean age of individuals diagnosed with CSC was 48.2 years with an overall peak prevalence in the age group 41–50 years. This is consistent with the existing literature, which describes a mean age ranging between 41 years (Kitzmann et al., 2008) and 55.5 years (Lange et al., 2024). It is important to stress that CSC has been reported in individuals under the age of 18 as well as among the elderly (Lange et al., 2024; Spaide et al., 1996; Velazquez‐Martin et al., 2012). In our study, we also report a small number of cases in both young individuals aged 11–20 years as well as elderly individuals aged 71–80 years. However, due to the fundus photography‐based approach, we speculate that we may be underestimating the number of cases in these age extremes, as the cause of subretinal fluid in these age categories is less likely to be due to CSC.

Limitations of this study need to be acknowledged to understand the accuracy of its conclusions. First, for each single individual, only one action diagnosis can be obtained in this tele‐ophthalmological system. Thus, data are simplified to the most severe/action requiring diagnosis when considering individuals with multiple diagnoses in one eye or different diagnoses in each eye. This approach will theoretically neglect more benign cases of CSC and underestimate the true prevalence. Second, the diagnosis of CSC was based purely on the tele‐ophthalmological evaluation. Therefore, cases of CSC detected are likely to be those with active CSC and those that are clearly visible on colour fundus photography. The tele‐ophthalmological diagnosis was not supplemented by slit‐lamp biomicroscopy, optical coherence tomography or retinal angiography, which would have improved the accuracy of the diagnosis. This source of bias is likely to underestimate the true prevalence of CSC. Third, we do not have data to investigate potential false negatives among those who were examined by an optometrist but never referred to the tele‐ophthalmological evaluation. A potential high number of false negatives, for example due to minimal symptoms or very subtle/no sign on fundus photography, would lead to an underestimation of the prevalence of CSC. Finally, opportunistic examination inherently leads to a selection bias, as it is more likely to examine individuals with symptoms than those without symptoms. Since the opportunistic examination in this study occurred in optician stores, it is also more likely for individuals with known refractive errors to be examined than those without. In that regard, hyperopia and short axial length are known risk factors of CSC (Terao et al., 2020), whereas myopia is a known protective factor of CSC (Manayath et al., 2016). Also, it can be speculated whether individuals with active CSC will seek optometrist evaluation when they are aware that they have a disease or if they have been advised to wait until the fluid is resolved before seeking optical products (Schou et al., 2024). These circumstances complicate the accuracy of our finding.

In conclusion, this large epidemiological study examined one in six of the entire population of Denmark and found that the prevalence of CSC was 14 per 100 000. We found that male biological sex is also a significant risk factor in a Nordic/Scandinavian population. Further studies employing multimodal imaging are warranted, including the use of macular optical coherence tomography.

## CONFLICTS OF INTEREST STATEMENT

D.V.M. declares to have received consultancy honorarium from Alcon. J.G. declares to have received speaker's fee from Allergan, Bayer, Novartis and Roche, and to have served as an advisory board member for Allergan, Apellis, Bayer, Novartis and Roche, not related to this work. M.S.H. declares speaker's fee for Roche. M.L.R.R. declares to have received speaker's fee for lectures from Santen, and to have received advisory board honorarium from Santen. Y.S. declares to have received speaker's fee for lectures from Bayer and Roche. All other authors declare that no potential conflicts of interest exist in relation to this work.

## ETHICS STATEMENT

This study followed the tenets of the Declaration of Helsinki. All participants provided mandatory consent to data processing and company policies. Data storage and management complied with the standards of the European Union General Data Protection Regulation. Data extracted were fully anonymous and are classified as quality assessment; hence, neither ethics approval nor any permission for the use of the data were needed according to The Danish Data Protection Agency.
